# Atherogenic index of plasma is an independent predictor of metabolic-associated fatty liver disease in patients with type 2 diabetes

**DOI:** 10.1186/s40001-022-00731-x

**Published:** 2022-07-11

**Authors:** Sahar Samimi, Sahar Rajabzadeh, Soghra Rabizadeh, Manouchehr Nakhjavani, Pooria Nakhaei, Foroogh Alborzi Avanaki, Alireza Esteghamati

**Affiliations:** 1 Endocrinology and Metabolism Research Center (EMRC), Tehran University of Medical Sciences, Vali-Asr Hospital Complex, Tohid Square, Tehran, 1419733141 Iran; 2 Department of Gastroenterology and Hepatology, Tehran University of Medical Sciences, Tehran, Iran

**Keywords:** Atherogenic index of plasma, Non-alcoholic fatty liver disease, Metabolic-associated fatty liver disease, Diabetes mellitus

## Abstract

**Background:**

Metabolic-associated fatty liver disease (MAFLD), formerly known as non-alcoholic fatty liver disease, is the leading cause of liver disease that can ultimately lead to cirrhosis. Identifying a screening marker for early diagnosis of MAFLD in patients with type 2 diabetes (T2D) can reduce the risk of morbidity and mortality. This study investigated the association between the atherogenic index of plasma (AIP) and MAFLD in patients with T2D.

**Method:**

A retrospective case–control study was conducted and medical records of patients with T2D were assessed. The baseline characteristics, anthropometric indices, laboratory measurements including liver functions tests, fasting blood sugar, HbA1C, lipid profile were documented.

**Results:**

Out of 2547 patients with T2D, 824 (32.4%) had MAFLD. The multivariate logistic regression analysis showed a significant difference in female-to-male ratio (1.11 vs. 1.33, OR = 0.347, *P*-value < 0.001), ALT (42.5 ± 28.1 vs. 22.4 ± 11.1, OR = 1.057, *P*-value < 0.001), and AIP (0.6 ± 0.3 vs. 0.5 ± 0.3, OR = 5.057, *P*-value < 0.001) between MAFLD and non-MAFLD groups, respectively. According to the AIP quartile, the prevalence of MAFLD increased significantly in patients with higher AIP quartiles (*P*-value < 0.001). Also, we found a cut-off of 0.54 for AIP in predicting MAFLD in patients with T2D (sensitivity = 57.8%, specificity = 54.4%).

**Conclusion:**

In this study, we found that AIP is a good and independent predictor for MAFLD in patients with T2D which could help physicians in early diagnosis and follow-up of patients with T2D.

## Background

Non-alcoholic fatty liver disease (NAFLD) is described as hepatic fat accumulation demonstrated either by imaging or histology, without the presence of significant alcohol consumption or other secondary causes of steatosis [1–4]. Based on previous studies, the NAFLD incidence rate in patients with type 2 diabetes (T2D) is approximately 2 times higher than the general population (50 to 75%, as opposed to 25%, respectively) [5–7]. Coexistence of both NAFLD and T2D is highly important for clinicians, as it not only leads to higher micro- and macro-vascular diabetes complications, but also increases the risk of NAFLD progression to cirrhosis, hepatocellular carcinoma, and death [8].

It is worth noting that international experts have recently proposed the novel term metabolic-associated fatty liver disease (MAFLD), instead of NAFLD. As the pathophysiology leading to NAFLD has become clearer, a new set of “positive criteria” has been recommended, instead of a mere exclusion of other diagnoses. The proposed criteria for MAFLD include one of the following three criteria: overweight/obesity, T2D or evidence of metabolic dysregulation, in addition to hepatic steatosis [9].

Considering the aforementioned studies, early diagnosis of MAFLD in T2D plays a major role in reducing patient morbidity and mortality. In this regard, markers such as liver enzymes and BMI lack sensitivity as screening markers [10, 11]. However, atherogenic index of plasma (AIP), defined as the logarithm of triglyceride-to-high density lipoprotein cholesterol ratio (TG/HDL-C) [12], may overcome limitations, and possibly be useful as a tool for MAFLD screening and follow-up. A significant correlation between AIP and NAFLD has previously been found [10, 11, 13]; however, to the best of the authors’ knowledge, the association between AIP and MAFLD/NAFLD in T2D patients has not been previously investigated.

Previous studies have represented AIP as a good predictor for T2D [14–16]. Additionally, multiple studies have demonstrated that higher levels of AIP were associated with various micro- and macrovascular complications of diabetes including coronary artery disease, metabolic syndrome, nephropathy, and neuropathy [12, 14, 17–21].

The main objectives of the present study were to investigate the relationship between AIP and MAFLD in T2D patients, and figure out whether AIP can be used as an independent biomarker to predict MAFLD in T2D individuals and facilitate early diagnosis in this specific population.

## Materials and methods

### Study population

The subjects of this case–control study were recruited at the diabetes clinic of Vali-Asr hospital, affiliated with Tehran University of Medical Sciences. The inclusion criteria were a diagnosis of T2D based on the 2021 American Diabetes Association guideline [22]. The exclusion criteria were age < 18, type 1 diabetes, history of malignancy, heart failure and cirrhosis. A total of 2547 patients with T2D were enrolled and divided into two groups: patients with and without MAFLD.

### Data collection

Patients’ baseline characteristics such as age, gender, duration of diabetes, height, weight, waist and hip circumferences, systolic and diastolic blood pressure, and laboratory measurements including fasting blood glucose (FBS), hemoglobin A1c (HbA1c), low-density lipoprotein (LDL), high-density lipoprotein (HDL), triglyceride (TG), aspartate aminotransferase (AST), alanine aminotransferase (ALT), alkaline phosphatase (ALP), gamma-glutamyl transferase (GGT), creatinine and history of anti-lipid therapy (i.e., statins and fibrates) were extracted from medical records on their first visit.

Anthropometric indices were retrieved by eligible medical staff. Waist and hip circumferences were measured horizontally at the level of the umbilicus and the widest part of the buttocks, respectively, while the patient was standing. For body mass index (BMI) calculation, weight was measured in kilograms and height in meters and calculated as weight divided by the square of height. Blood pressure was measured with an automatic blood pressure device after 15 min rest upon arrival. The average of two blood pressure recordings, retrieved 10 min apart, was recorded. GFR was calculated by the Modification of Diet in Renal Disease (MDRD) equation.

All blood samples were collected after a minimum of 10–12 h overnight fasting and evaluated with kits approved by the central reference laboratory. HbA1c was measured via high-performance liquid chromatography (A1C, DS5 Pink kit; Drew, Marseille, France). FBS was measured by enzymatic calorimetry methods with the glucose oxidase test and serum lipid indices (TG, HDL, LDL) were measured using enzymatic methods.

AIP was calculated as the logarithmic transformation of the triglyceride-to-HDL cholesterol ratio. Regarding ultrasonography criteria for NAFLD, diagnosis was made when at least two of three findings were reported by a trained radiologist: diffusely echogenic liver (known as “bright liver”), vascular blurring, and narrowing of the hepatic veins [23].

### Statistical analysis

All statistical analyses were performed with the SPSS software version 25 for Windows and a value of *P*-value < 0.05 was considered statistically significant. We used Kolmogorov–Smirnov and Shapiro–Wilk normality tests, P–P plot, and histogram to confirm the study population’s normal distribution.

Continuous variables were presented as means ± standard deviations (SD) for variables with normal distribution and median and interquartile range for variables without a normal distribution. These variables were compared between patients with and without MAFLD using the *T*-test. For categorical variables, characteristics were recorded as frequencies or percentages and Chi-square analysis was performed to assess the relationship with MAFLD.

Multivariate logistic regression analysis was conducted to evaluate the association between AIP and other indices with MAFLD. Odds ratios (ORs) were retrieved from logistic regression analysis, and presented with a 95% confidence interval (CI). Area under the curve (AUC) of receiver operating characteristic (ROC) was calculated to define the predictive value of AIP for MAFLD in patients with diabetes and the cut-off for AIP was calculated via the Youden index.

## Results

A total of 2547 subjects with T2D took part in this study, divided in to 824 (32.4%) MAFLD patients and 1723 (67.6%) non-MAFLD controls. Table 1 compares the baseline characteristics of the two groups of patients with T2D. The mean age of non-MAFLD patients was 60.24 ± 10.80 and 57.05% (983) of them were female. The mean age of MAFLD controls was 54.7 ± 11.43 and 52.7% (434) of them were female.

**Table 1:** Comparisons of baseline characteristics of patients with T2D with and without MAFLD

	Non-MAFLD *N* = 1723	MAFLD *N* = 824	*P*-value
Age, years	60.24 ± 10.80	54.70 ± 11.43	< 0.001
Gender, %(*N*)			
Female	57% (983)	52.66% (434)	0.022
Male	43% (741)	47.33% (390)	
Duration of DM, years	10.87 ± 8.60	9.55 ± 7.35	< 0.001
SBP, mmHg	133.37 ± 36.24	132.94 ± 76.45	0.877
DBP, mmHg	78.22 ± 9.90	79.49 ± 7.52	0.001
BMI, kg/m^2^	28.60 ± 5.10	30.65 ± 5.10	< 0.001
Waist/hip	0.94 ± 0.22	0.94 ± 0.06	0.456
eGFR, mL/min/1.73 m^2^	79.11 ± 32.15	90.85 ± 36.34	< 0.001
Microalbuminuria	19.5% (230)	17.1% (92)	0.126
HOMA-IR	3.37 ± 3.03	4.74 ± 3.09	< 0.001
HDL, mg/dl	45.14 ± 11.90	43.88 ± 11.40	0.010
Non-HDL, mg/dl	135.63 ± 41.57	143.52 ± 43.40	0.003
LDL, mg/dl	95.43 ± 33.69	103.04 ± 34.35	< 0.001
TG, mg/dl	165.93 ± 93.22	189.03 ± 112.52	< 0.001
HbA1C, %	8.32 ± 1.752	7.40 ± 1.53	0.031
FBS, mg/dl	160.72 ± 57.31	152.82 ± 51.83	0.001
AST, U/L	19.69 ± 10.95	29.97 ± 18.31	< 0.001
ALT, U/L	22.36 ± 11.10	42.45 ± 28.07	< 0.001
ALKP, U/L	150.29 ± 74.89	167.60 ± 90.80	< 0.001
GGT, U/L	21 (16,33)	30.7 (20,40.25)	0.013
AIP	0.52 ± 0.25	0.59 ± 0.26	< 0.001
Anti-lipid therapy			
+	78% (1343)	65.8% (542)	< 0.001
−	22% (380)	34.2% (282)	

*MAFLD* metabolic-associated fatty liver disease. Age, duration of diabetes mellitus, waist/hip, *SBP* systolic blood pressure, *DBP* diastolic blood pressure, *eGFR* estimated glomerular filtration rate, *BMI* body mass index, *HBA1C* hemoglobin A1C, *HOMA-IR* homeostatic model assessment of insulin resistance, *HDL* high-density lipoprotein, non-HDL, *LDL* low-density lipoprotein, *TG* triglyceride, *FBS* fasting blood glucose, *AST* aspartate transaminase, *ALT* alanine transaminase, *ALP* alkaline phosphatase, *AIP* atherogenic index of plasma are presented as mean ± standard deviation. Microalbuminuria and gender are presented as percentage (frequency). *GGT* gamma-glutamyl transferase is presented as median (IQR)

As seen in Table 1, participants with T2D and MAFLD were more likely to be younger, to receive anti-lipid therapy and to be female (*P*-values < 0.001, < 0.001 and 0.022, respectively). They also had a significantly higher BMI (*P*-value < 0.001), eGFR (*P*-value < 0.001), DBP (*P*-value = 0.001), HOMA-IR index (*P*-value < 0.001), triglyceride (*P*-value < 0.001), LDL-c (*P*-value < 0.001), non-HDL-c(*P*-value = 0.003), AST (*P*-value < 0.001), ALT (*P*-value < 0.001), ALKP (*P*-value < 0.001), GGT (*P*-value = 0.013), AIP (*P*-value < 0.001), and a significantly lower HDL-c (*P*-value = 0.010), FBS (*P*-value = 0.001), HbA1C (*P*-value = 0.031), and duration of diabetes (*P*-value < 0.001). SBP (*P*-value = 0.877), waist–hip ratio (*P*-value = 0.456), and presence of microalbuminuria (*P*-value = 0.126) did not differ significantly between T2D patients with and without MAFLD.

Table 2 illustrates the frequencies and percentage of MAFLD and T2D patients according to the AIP quartile. In the first and fourth AIP quartile 26.8% (169) and 38.3% (237) of patients with T2D had MAFLD, respectively. The prevalence of MAFLD increased significantly in patients with T2D in higher AIP quartiles (*P*-value < 0.001).

**Table 2:** Prevalence of MAFLD in four groups according to AIP quartile in patients with T2D

AIP quartile	Non-MAFLD	MAFLD	*P*-value
1.00 (−0.53, 0.3760)	461	169	< 0.001
	73.2%	26.8%	
2.00 (0.3761, 0.5384)	449	174	< 0.001
	72.1%	27.9%	
3.00 (0.5385, 0.7117)	395	224	< 0.001
	63.8%	36.2%	
4.00 (0.7118, 1.53)	382	237	< 0.001
	61.7%	38.3%	

*AIP* atherogenic index of plasma, *MAFLD* metabolic-associated fatty liver disease

After adjustment for multiple confounders including gender, age, BMI, SBP, DBP, HbA1C, AST, ALT, ALKP, GGT, HOMA-IR and anti-lipid therapy in a multivariable logistic regression model, AIP showed an independent significant relationship with MAFLD in patients with T2D with an odd's ratio of 5.057 (*P*-value < 0.001). Table 3 represents the calculated odds ratios and *P*-values.

**Table 3:** Results of multivariate logistic regression analysis

	Beta	Standard error	Odds ratio	95% CI		*P*-value
				Lower	Upper	
AIP	1.621	0.441	5.057	2.133	11.991	< 0.001
Age, years	− 0.018	0.011	0.982	0.961	1.003	0.096
Gender (male)	− 1.052	0.251	0.347	0.212	0.567	< 0.001
Duration of DM, years	− 0.034	0.015	0.967	0.939	0.996	0.026
SBP, mmHg	0.000	0.001	1.000	0.997	1.002	0.909
DBP, mmHg	− 0.001	0.015	0.999	0.970	1.029	0.949
Waist/hip	− 0.358	1.619	0.699	0.029	16.698	0.825
HOMA-IR	0.164	0.054	1.178	1.059	1.311	0.003
FBS, mg/dl	0.000	0.003	1.000	0.995	1.005	0.868
AST, U/L	− 0.028	0.019	0.973	0.938	1.009	0.142
ALT, U/L	0.055	0.011	1.057	1.034	1.080	< 0.001
ALKP, U/L	− 0.003	0.001	0.997	0.995	1.000	0.039
GGT, U/L	0.006	0.004	1.006	0.999	1.014	0.099
Anti-lipid therapy ( +)	− 0.312	0.235	0.732	0.461	1.160	0.184

*AIP* atherogenic index of plasma, *SBP* systolic blood pressure, *DBP* diastolic blood pressure, *HOMA-IR* homeostatic model assessment of insulin resistance, *HDL* high-density lipoprotein, *FBS* fasting blood glucose, *AST* aspartate transaminase, *ALT* alanine transaminase, *ALP* alkaline phosphatase, *GGT* gamma-glutamyl transferase, *anti-lipid therapy ( +)* patients received anti-lipid therapy

**Fig. 1:**
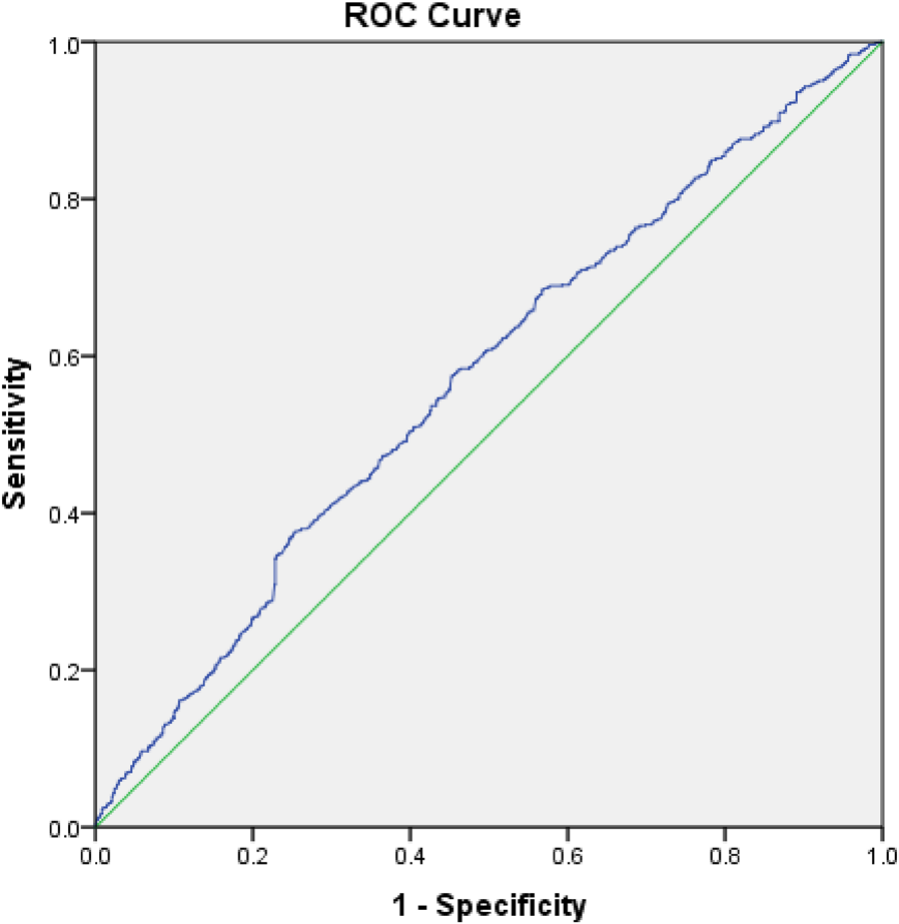
AUROC curve for AIP

In addition, Fig. 1 and Table 4 show the predictive ability of AIP for MAFLD diagnosis (AUC = 0.570, 95% CI 0.546–0.594, *P* < 0.001) and propose an AIP cut-off of 0.54 to predict MAFLD in patients with T2D (sensitivity = 57.8%, specificity = 54.4%).

**Table 4:** Multivariate logistic regression analysis

	AUC	95%CI	Sensitivity	Specificity	Cut-off
AIP	0.57	0.54–0.59	57.8%	54.4%	0.54

*AIP* atherogenic index of plasma, *AUC* area under the curve, *AUROC* area under the receiver operating characteristic

## Discussion

In this study, the relationship between AIP and MAFLD in patients with T2D was analyzed, with the intention to clarify whether this biomarker has predictive value for MAFLD in these patients. The results demonstrated that independent of age, sex, duration of diabetes, history of anti-lipid therapy, HOMA-IR, FBS, liver enzymes and blood pressure, patients with T2D were 5 times more likely to have a higher AIP with a proposed cut-off of 0.54. Findings from the current study suggested that AIP has better predictive value compared to markers such as liver enzymes (AST, ALT and ALP) and BMI, which were historically presumed to predict NAFLD in patients with diabetes.

Prompt diagnosis of MAFLD in patients with T2D, using a biomarker like AIP, is critical both due to the adverse outcomes that late diagnosis harbors, and because of the emerging pharmacotherapeutic interventions targeting MAFLD [24, 25]. Several observational studies have described an independent association between microvascular complications, such as chronic kidney disease and distal/autonomic neuropathy, and MAFLD [26–28]. The relationship between retinopathy and MAFLD, however, remains conflicting with limited available studies [24]. Regarding macrovascular complications, cardiovascular disease remains the most common cause of death among patients with MAFLD, and previous studies have demonstrated that patients with MAFLD are at a higher risk for CVD events, despite adjusting for relating confounders [24]. On the other hand, in patients with T2D, MAFLD progresses to cirrhosis at a much faster pace [29]. The abovementioned evidence, although based on observational studies that only illustrate associations and not causality, supports the crucial nature of early MAFLD diagnosis. On top of that, novel therapies such as dapagliflozin, aramchol, resmetirom, semaglutide, and lanifibranor have currently entered phase III clinical trials, opening up new horizons in curing MAFLD [25].

Although the accuracy of utilizing AIP to predict MAFLD in patients with T2D was not remarkable in the present study (AUROC of 0.57), this biomarker still remains potentially beneficial in clinic and epidemiological studies for the following reasons. Due to the skyrocketing costs of health care, measuring AIP which is both cheap and readily available, can be favorable. Additionally, the imperative nature of early diagnosis of MAFLD in patients with T2D, mentioned previously, adds to the value that AIP testing brings to the table.

To the best of the authors’ knowledge, the relationship between AIP and MAFLD in patients with T2D has not been investigated. However, previous studies have shown a significant correlation between AIP and NAFLD in the general population [12, 15]. Xie et al. who analyzed the relationship among various biomarkers and NAFLD in the general Chinese population, found that subjects with NAFLD were 14 times more likely to have a higher AIP. Their study demonstrated that AIP was a stronger predictor of NAFLD compared to previous biomarkers used to estimate the risk of NAFLD in patients with T2D such as liver enzymes (AST, ALT and ALP) and BMI [10]. Wang et al., studied the same markers in obese patients without diabetes. Their study also showed a significant association between AIP and NAFLD in obese participants with an odds ratio of 5.37 [11]. Similarly, Dong et al., studied AIP levels specifically in non-obese Chinese and Japanese participants, illustrating that AIP was the strongest factor positively correlating with NAFLD with a cut-off of 0.005 for subjects with Chinese ethnicity and − 0.220 in the Japanese group. Patients with NAFLD in their study were approximately 15 times more likely to have a high AIP, as opposed to raised liver enzymes and BMI [13]. All three previous studies were performed on patients regardless of their history of diabetes.

Previous research has illustrated a negative correlation between anti-lipid therapy and AIP level [24]. Therefore, compared to the aforementioned studies [10, 11, 13], we went one step further and included anti-lipid therapy as a confounding factor in our analysis and still managed to demonstrate a high odds ratio.

Our findings although compatible with previous studies in showing the role of AIP in predicting NAFLD, differed in the best cut-off for AIP. Firstly, when interpreting these results, the impact of race on lipid profiles and therefore AIP should be kept in mind. In a large cohort study, Giannini et al. found interethnic variations in the triglyceride-to-HDL ratio among the obese youth of African-Americans, Hispanics and White decent [31]. In a comprehensive study by Frank et al. on dyslipidemia among ethnic groups, most minority groups had higher TGs compared to non-Hispanic-Whites [32], and Huxley et al., identified that isolated low HDL-c is more common among Asians compared to non-Asians [33]. In Dong et al.’s study, the cut-off of for AIP in predicting NAFLD in the subjects with Chinese ethnicity was 0.005 as opposed to − 0.220 in the Japanese group [13]. These findings all suggest that AIP may differ in various ethnic groups and may explain the observed differences in results from the present study compared to previous studies on the relationship between AIP and NAFLD.

Secondly, the present study’s subjects were specifically patients with T2D. Individuals with T2D commonly suffer from dyslipidemia presented as elevated triglycerides (TG), low HDL-cholesterol (HDL-C) levels and the predominance of small dense LDL (SD-LDL) particles [34, 35]. Additionally, multiple studies have shown that the ratio of TG/HDL-C and therefore AIP is positively correlated to insulin resistance (IR) [36]. This study represented that AIP was significantly higher in patients with T2D and MAFLD compared to patients with diabetes, without MAFLD. However, as patients with T2D already have a higher baseline AIP regardless of having MAFLD, our cut-off differed from previous studies that were performed on the general population [31–36].

In this study, we also witnessed that patients without MAFLD were more likely to have an abnormal glycemia profile illustrated by higher FBS and HbA1c, and a lower eGFR. One assumption is that patients with MAFLD were possibly more conscious of its health outcome; thus, they adhered to positive lifestyle modifications leading to better glycemia control and less complications.

The present study was performed on the Iranian population and proposes a cut-off of 0.54 for AIP to predict MAFLD in patients with T2D. The authors propose that further research should be done on the association between AIP and MAFLD in patients with T2D.

## Conclusion

In summary, our study showed that AIP was associated with MAFLD in patients with T2D. Therefore, AIP can be further investigated and potentially used as a predictive index for MAFLD in the follow-up of patients with T2D or as a target biomarker for MAFLD in diabetic patients enrolled in clinical trials.

## Data Availability

The datasets generated during and/or analyzed during the current study are available from the corresponding author on reasonable request.

## Abbreviations

NAFLD: Non-alcoholic fatty liver disease
MAFLD: Metabolic-associated fatty liver disease
T2D: Type 2 diabetes mellitus
AIP: Atherogenic index of plasma
FBS: Fasting blood sugar
HbA1c: Hemoglobin A1c
LDL: Low-density lipoprotein
HDL: High-density lipoprotein
TG: Triglyceride (TG)
AST: Aspartate aminotransferase
ALT: Alanine aminotransferase
ALP: Alkaline phosphatase
GGT: Gamma-glutamyl transferase
SBP: Systolic blood pressure
DBP: Diastolic blood pressure
eGFR: Estimated glomerular filtration rate
HOMA-IR: Homeostatic model assessment for insulin resistance
